# Impact of fixed orthodontic appliances on quality of life among adolescents’ in India

**DOI:** 10.4317/jced.51651

**Published:** 2014-10-01

**Authors:** Ramesh Nagarajappa, Gayathri Ramesh, Nagarajappa Sandesh, Ravishankar-Telgi Lingesha, Mohammed- Abid-Zahir Hussain

**Affiliations:** 1 MDS, Professor and Head. Department of Public Health Dentisty, Rama Dental College and Hospital, A-1/8, Lakhanpur, Kanpur – 208024, Uttar Pradesh, India; 2 MDS, Associate Professor. Department of Oral and Maxillofacial Pathology, Rama Dental College and Hospital, A-1/8, Lakhanpur, Kanpur – 208024, Uttar Pradesh, India; 3 MDS, Associate Professor. Department of Public Health Dentistry, Sri Aurobindo College of Dentistry, Indore, Madhya Pradesh, India; 4 MDS, Associate Professor. Department of Public Health Dentistry, Kothiwal Dental College and Hospital, Kanth Road, Moradabad, Uttar Pradesh, India; 5 MDS, Professor. Department of Prosthodontics, Taibah University, KSA

## Abstract

Context: Malocclusion can seriously impair quality of life and they may affect various aspects of life, including function, appearance, interpersonal relationships and even career opportunities. Objectives: To assess and determine various factors that may influence the impact of orthodontic treatment on the quality of life of adolescents. Study Design: Cross sectional study in adolescents of Moradabad was conducted on 109 males and 113 females (n=222) adolescents having a fixed orthodontic appliance, aged 13 to 22 years (mean 17.5±1.5). A pre-structured questionnaire designed by Mandall et al, with nine conceptual impact sub-scales to highlight the problem faced by the patient in daily life after wearing the appliance was used to collect the data. Unpaired t-test was used to determine the statistical significance and the influence of variables were analysed using multiple linear regression analysis. Results: Factors which demonstrated high impact were oral hygiene (Mean=3.42; SD=0.78) followed by time constraints (Mean=3.23; SD=0.72) and physical impact (Mean=3.00; SD=0.61). Gender difference showed statistical significance in social impact (p=0.009), time constraints (p=0.001) and travel or cost implications (p=0.009). Internal reliability of the questionnaire ranged from low to good (Cronbach’s alpha 0.29-0.81). Test-retest reliability ranged from an intra-class correlation coefficient 0.09-0.42. Conclusions: Patients who had been comprehensively informed about their treatment had greatest levels of satisfaction and compliance with treatment. Younger patients showed an earlier adaptation to treatment with fixed appliances which influenced the treatment to be started at the earliest possible age.

** Key words:**Impact, malocclusion, quality of life.

## Introduction

Aesthetics has become an important issue in modern society as it seems to define one’s character. In the past, functional demand was the main consideration in the dental treatment while today the focus has shifted towards dental aesthetics. Social and cultural expectations and pressures produce a culturally valid need for orthodontic treatment (1). In the field of orthodontics there is a long standing recognition that malocclusion and dentofacial anomalies can produce immense physical, social and psychological upset. Increasingly patient centred measures are used to assess the orthodontic need and in determining the outcomes of orthodontic care (2).

Quality of life is an increasingly important component of the evaluation of treatment outcomes and has been defined as the discrepancy between our expectations and experiences. Oral diseases seriously impair quality of life in a large number of individuals and they may affect various aspects of life, including function, appearance, interpersonal relationships and even career opportunities (3). There is little research regarding orthodontic treatment in relation to health related quality of life. Clinicians are expected to be accountable for the effectiveness of the treatment and efficient use of resources (4). In orthodontics, health related quality of life issues have been developed and discussed in relation to adults undergoing orthognathic surgery (3).

Patients presenting with severe dentofacial deformities may require a comprehensive orthodontic and surgical approach to their treatment [so-called orthognathic treatment]. This treatment involves a course of fixed orthodontic appliances followed by surgery to correct the skeletal discrepancy which may extend upto 2 years for completion. These patients tend to be in the younger age group and currently lacking is any instrument to determine changes in quality of life as a result of this mode of treatment (5).

Previous studies have measured patient and parents expectations during orthodontic treatment, which introduces bias into the results. Another study measured patient’s expectations of pain resulting from wearing fixed ortho appliances, while the general expectations of ortho treatment were not investigated (1).

A measure of the impact of fixed appliances on daily life would be a useful way of highlighting problems that patient experiences. This is particularly true since it was concluded that there is a lack of information on the patient experiences and if patients were armed with adequate knowledge, this may possibly reduce some anxiety (6). In turn, we should be able to identify areas where patient may be pre-warned of specific potential problems as patient generally felt that they had a lack of satisfactory information prior to fitting their appliances (7). It was found that patients who had been comprehensively informed about their treatment had greatest levels of satisfaction and compliance with treatment (8).

The significant lack of and need for social indicators and a comprehensive approach to measuring the social and psychologic impacts of dental disease has been highlighted in several recent reports (9). So the present study was designed to assess the various factors and to determine the impact of orthodontic treatment on the quality of life of adolescents.

## Material and Methods

The study population consisted of 222 [109 males and 113 females] adolescents aged 13 to 22 years with the mean age of 17.5±1.5 years having a fixed appliance [orthodontic]. Sample size estimations indicated a sample larger than 180 when alpha is 0.05 and power is 0.80.

Ethical approval was granted by Kothiwal Dental College and Research Centre Ethics Committee. Informed consent was obtained from the child and the parent[s]. Patients were selected from the Department of Orthodontics at Kothiwal Dental College and Research Centre, Moradabad as they completed an adjustment appointment. All the study subjects were interviewed regarding the impact of orthodontic appliance on the quality of life over time at the first, second and third visits after their fixed appliance had been placed. They, thus, gave viewpoints relevant to all stages of fixed appliance treatment.

The interviewer used a pre-structured questionnaire with nine conceptual impact sub-scales: aesthetic, functional limitation, dietary impact, oral hygiene impact, maintenance impact, physical impact, social impact, time constraints and travel/cost implications to highlight the problem faced by the patient in daily life after wearing the ortho appliance designed by Mandall *et al*, 2006. Each interview took approximately 15 minutes to complete. The response options for the questions were on a Likert scale of 1-5 where 1= strongly disagree, 2= disagree, 3= neither agree nor disagree, 4= agree, 5= strongly agree.

Inclusion criteria for participation in the study were:

- Patients aged 13-22 years;

- Consent obtained from both the child and the parent.

- Patients not having any craniofacial anomalies such as cleft lip and palate.

The questionnaire was pre tested on ten patients who commented on its clarity, phrasing, simplicity and understanding. Relevant changes were made to some questions based on their response and then pre-piloted on a further ten patients. This helped to ensure that the questionnaire was not too long and patients did not have difficulty with any sections.

Internal consistency of the questionnaire was assessed using Cronbach’s alpha. The test-retest reliability was assessed using intra-class correlation coefficients. The unpaired t-test was used to determine the statistical significance in the overall scores. Multiple stepwise linear regression analysis was used to evaluate the influence of age, sex and place on the impact of fixed orthodontic appliance. The *p*-value of 0.05 or less was considered as statistically significant.

## Results

The reliability of the questionnaire including nine subscales to assess the impact of fixed appliances on daily life is shown in  Table 1. The internal consistency was estimated and ranged from moderate to good [Cronbach’s alpha ≥ 0.60 for all subscales except for social impact [0.5938] and maintenance impact [0.5131]. It was poor in relation to functional limitations [0.2982]. Test-retest reliability analysis was performed by comparing the two sets of scores for each component using intra-class correlation coefficient which ranged from 0.0946 to 0.4271.

**Table 1:**
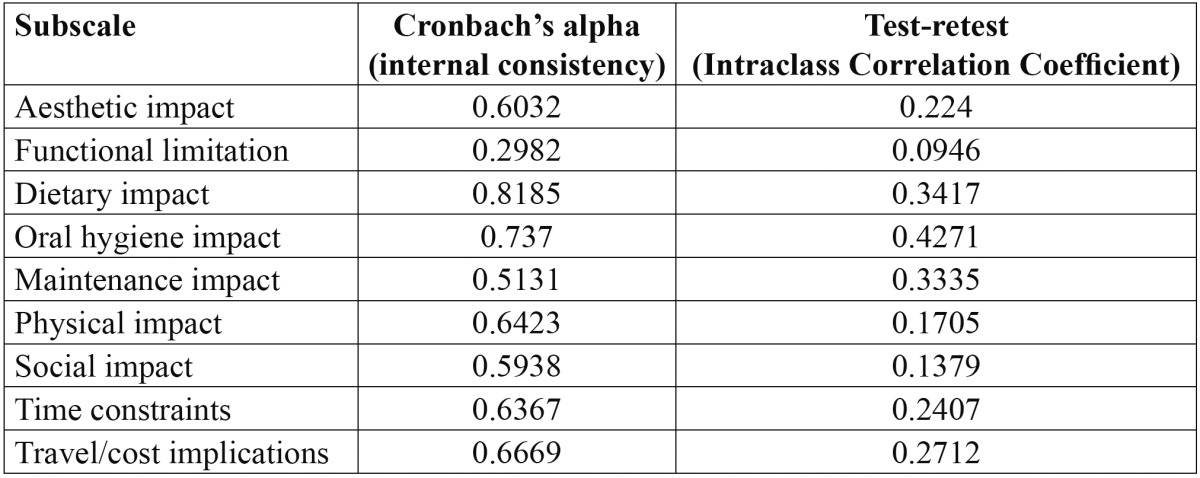
The reliability of the questionnaire subscales to assess the impact of fixed appliances on daily life.

 Table 2 shows the impact of fixed orthodontic appliances on adolescents’ quality of life in relation to different factors. Subjects self-reported estimation revealed oral hygiene [Mean=3.42; SD=0.78] followed by time constraints [Mean=3.23; SD=0.72] and physical impact [Mean=3.00; SD=0.61] to have a major influence on their quality of life.

**Table 2:**
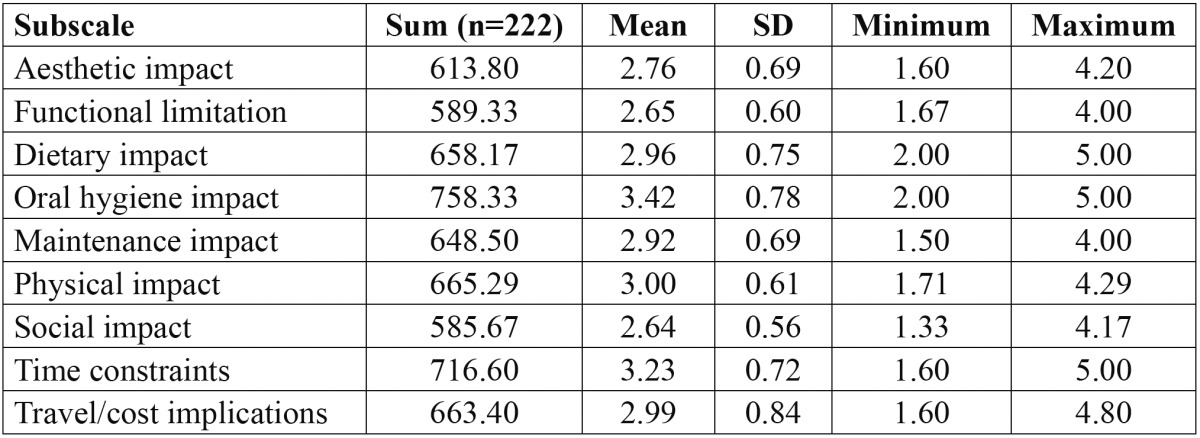
The overall impact of different factors on quality of life among adolescents’ with fixed orthodontic appliances.

 Table 3 shows the comparison of mean responses and standard deviations in between males and females. The only subscales which had significantly influenced the quality of life when assessing the gender difference were social impact [*p*=0.009], time constraints [*p*=0.001] and travel or cost implications [*p*=0.009].

**Table 3:**
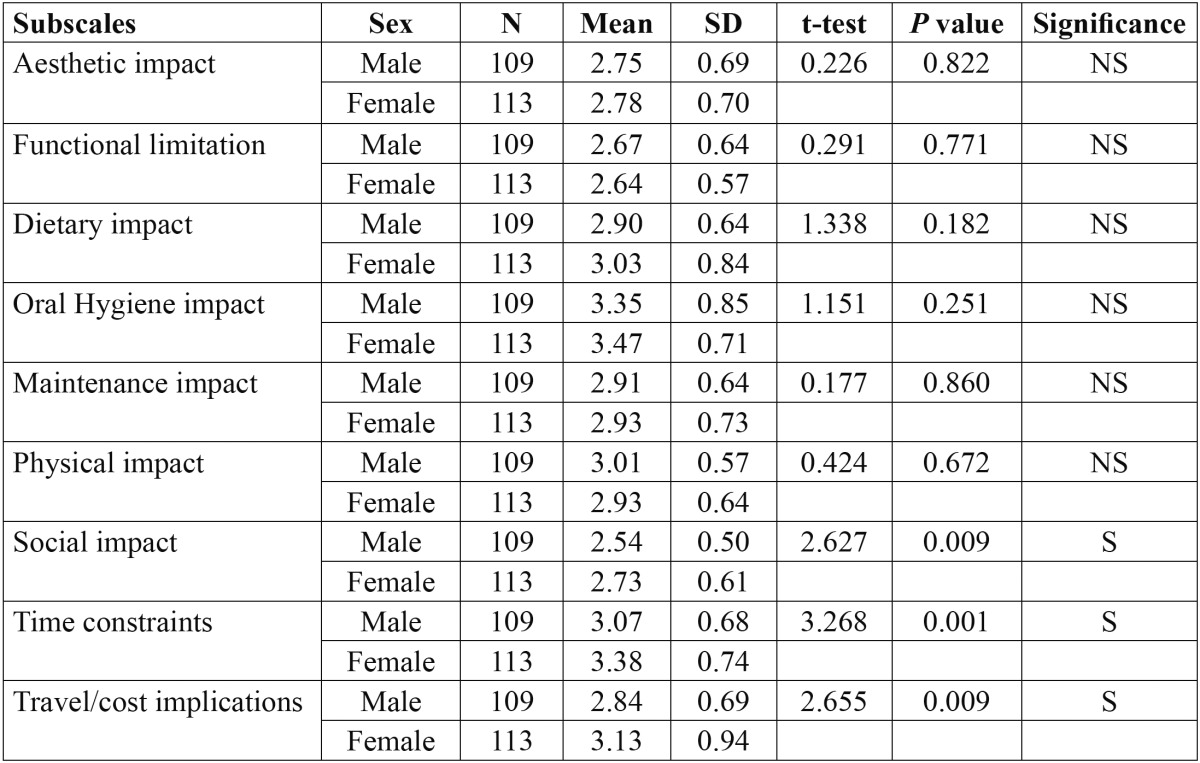
Comparison of mean responses and the impact of fixed orthodontic appliances on quality of life according to sex.

Similarly all the factors were compared in relation to places, Moradabad proper and places situated within 50 kms from Moradabad ( Table 4). The analysis revealed a statistically significant impact of fixed orthodontic appliances, with respect to dietary impact [*p*=0.003], oral hygiene impact [*p*=0.000], maintenance impact [*p*=0.036], physical impact [*p*=0.001], time constraints [*p*=0.013], and travel/cost implications [*p*=0.000].

**Table 4:**
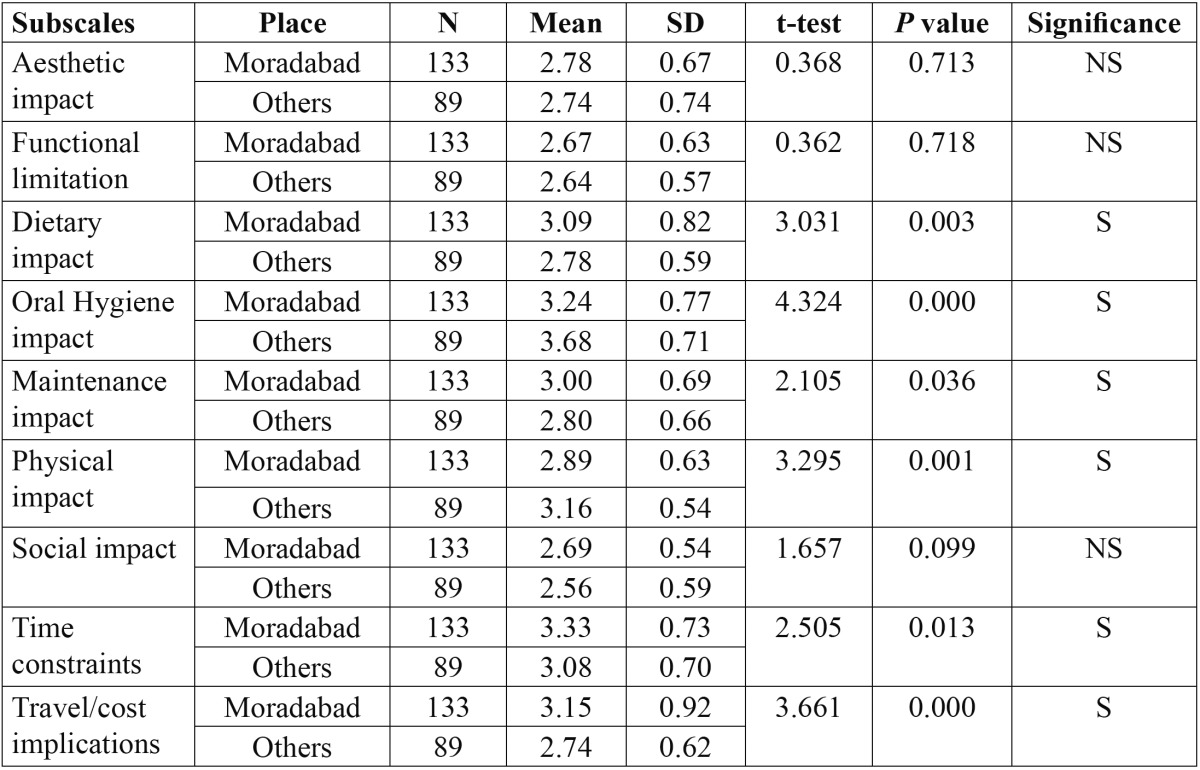
Comparison of mean responses and the impact of fixed orthodontic appliances on quality of life according to place.

Multiple linear regression analysis revealed the influence of age, sex and place on the impact of fixed appliances on daily life. All the independent variables had an influence on the impact of fixed appliances which was fairly low and was also found to be statistically significant. Generally, patients who were young, females and those belonging to the same place were less affected by their fixed appliances based on the responses ( Table 5).

**Table 5:**
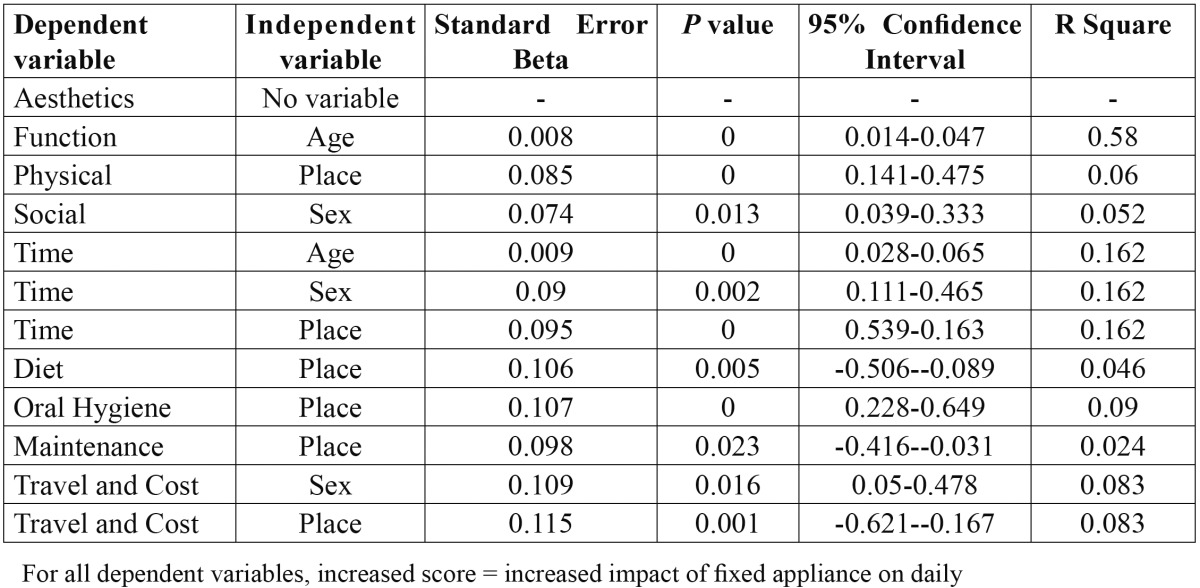
Multiple linear regression analysis investigating any influence of age, sex and place on the impact of fixed appliances on daily life.

## Discussion

Within the field of orthodontics there is a long standing recognition that malocclusion and dentofacial anomalies can produce immense physical, social and psychological upset. Greater understanding of patients’ expectations of the orthodontic treatment process and how it affects their day-to-day living or quality of life [QoL] is important in many ways. Their expectations of treatment, unrealistic understanding of orthodontic treatment processes and sequelae can influence compliance with treatment (2).

It was surprising to find the impact of all the subscales to be fairly low in our study. A possible explanation for this, in terms of discomfort, is that patients may expect some degree of pain from their appliance and can therefore cope with it more effectively. Alternatively, since post-adjustment pain is relatively short-lived, between four and 24 hours depending on patient age (10), the overall impact on daily life in-between appointments may be lower than expected.

Although this questionnaire did not measure the impact of dental disease, the domains or subsets of questions emerging were similar to previously published literature (11). When a patient’s smile is destroyed by dental disease, the result often is loss of self-esteem and damage to his or her overall physical and mental health (12). The social and cultural expectations with regard to the dental appearances have changed with times due to the widespread use of orthodontic services and acceptance of treatment of malocclusion.

The minimal dietary impact may be due to;

• Some children eating softer diets than others, even before the appliance fitting.

• Clinical experience suggests that some children ignore advice about avoiding hard foods and the impact on their dietary habits may be low.

• Some patients may simply carry on eating the harder foods but modify their methods, for example cutting food into smaller pieces. In contrast, other children may simply avoid the harder foods but miss them more.

Maintenance impact of broken appliances also had a low test-retest correlation coefficient and this may likely be due to the relatively low appliance fracture rate among the study participants. Patients having no experience of a broken appliance found this question difficult and if they had broken their appliance only at the second repeat questionnaire, their response would be unreliable, compared with their response in the first questionnaire. Thus, many patients might have answered this question from a theoretical or imaginary viewpoint.

The effect of age, sex, place on the impact of fixed appliances.

Generally, age was an influential variable on the daily impact of fixed appliances with younger patients appearing to cope better with their appliance. There is no literature with which to compare the effect of age on fixed appliance impact. However, younger patients have lower treatment discontinuation rates (13) and it may be hypothesized that this is because the impact of fixed appliances is lower in younger children, who may then cooperate better with treatment. In addition, the patients who had been comprehensively informed about their treatment had greatest levels of satisfaction and compliance with treatment (8).

Similarly, the influence of gender on impact of fixed appliances was also low. Dental esthetics was found to be more important among women than men who can be used to explain lower discontinuation rates for girls (14). Lastly, the data suggested that the impact of fixed appliances on travel and cost of attending was not affected by social deprivation. This may be due to the cost of attendance being spread over 1-2 years and is not perceived as a burden.

The possession of malocclusion has more impact on one’s emotional well-being than on actual dental health or function (15). It is generally accepted that the patients benefit from a psychological point of view with improved facial and dental appearance and the associated increased self-confidence which accompanies this. Patients and parents expected orthodontic treatment to improve mastication, speech and success in future occupations (16) along with an increase in social confidence (17).

It should be acknowledged that a criticism about well-being or quality of life may adapt or habituate people’s health conditions over time. Thus, the subjects may respond with lower impact scores when a questionnaire is re-administered at a later time. It was surprising that impact such as diet and oral hygiene did not reduce over time as patients become used to the appliance. Conversely, it may be expected that pain and discomfort do not diminish with time as the appliance is being regularly adjusted. However, as treatment progressed the components or subscales were significantly less compromised than anticipated which may reflect either actual decreases in the level of impact experienced, adaptation to treatment, or learned experience of treatment (18).

These estimated factors hold promise for assessing the more subtle ways in which fixed orthodontic appliance therapy impacts the quality of life. However, the relationship between parents’ occupation and socioeconomic status can also be explored in further studies.

## Conclusions

Dentofacial deformities seriously impair the individuals quality of life including function, appearance, interpersonal relationships and even career opportunities. Treatment should be started at the earliest possible age as younger patients are more adaptable to treatment with fixed appliances. The findings can be used to educate, reassure and motivate patients at the start of treatment.
